# A Zebrafish Model for *Chlamydia* Infection with the Obligate Intracellular Pathogen *Waddlia chondrophila*

**DOI:** 10.3389/fmicb.2016.01829

**Published:** 2016-11-18

**Authors:** Alexander G. J. Fehr, Maja Ruetten, Helena M. B. Seth-Smith, Lisbeth Nufer, Andrea Voegtlin, Angelika Lehner, Gilbert Greub, Philip S. Crosier, Stephan C. F. Neuhauss, Lloyd Vaughan

**Affiliations:** ^1^Vetsuisse Faculty, Institute for Veterinary Pathology, University of ZurichZurich, Switzerland; ^2^Functional Genomics Center Zurich, Molecular and Life Sciences, University of ZurichZurich, Switzerland; ^3^Vetsuisse Faculty, Institute of Veterinary Bacteriology, University of ZurichZurich, Switzerland; ^4^Vetsuisse Faculty, Institute for Food Safety and Hygiene, University of ZurichZurich, Switzerland; ^5^Institute of Microbiology, University Hospital Center and University of LausanneLausanne, Switzerland; ^6^Department of Molecular Medicine and Pathology, School of Medical Sciences, University of AucklandAuckland, New Zealand; ^7^Institute of Molecular Life Sciences, University of ZurichZurich, Switzerland

**Keywords:** zebrafish, PVC superphylum, *Chlamydia*, *Waddlia*, swim bladder infection, endothelial cells, neutrophils, MyD88

## Abstract

Obligate intracellular chlamydial bacteria of the Planctomycetes-Verrucomicrobia-Chlamydiae (PVC) superphylum are important pathogens of terrestrial and marine vertebrates, yet many features of their pathogenesis and host specificity are still unknown. This is particularly true for families such as the *Waddliacea* which, in addition to epithelia, cellular targets for nearly all *Chlamydia*, can infect and replicate in macrophages, an important arm of the innate immune system or in their free-living amoebal counterparts. An ideal pathogen model system should include both host and pathogen, which led us to develop the first larval zebrafish model for chlamydial infections with *Waddlia chondrophila*. By varying the means and sites of application, epithelial cells of the swim bladder, endothelial cells of the vasculature and phagocytosing cells of the innate immune system became preferred targets for infection in zebrafish larvae. Through the use of transgenic zebrafish, we could observe recruitment of neutrophils to the infection site and demonstrate for the first time that *W. chondrophila* is taken up and replicates in these phagocytic cells and not only in macrophages. Furthermore, we present evidence that myeloid differentiation factor 88 (MyD88) mediated signaling plays a role in the innate immune reaction to *W. chondrophila*, eventually by Toll-like receptor (TLRs) recognition. Infected larvae with depleted levels of MyD88 showed a higher infection load and a lower survival rate compared to control fish. This work presents a new and potentially powerful non-mammalian experimental model to study the pathology of chlamydial virulence *in vivo* and opens up new possibilities for investigation of other members of the PVC superphylum.

## Introduction

The bacterial species *Waddlia chondrophila* is a purported abortifacient pathogen of cattle (Dilbeck-Robertson et al., 2003), first isolated from a cow abortion in the United States (Dilbeck et al., 1990) and subsequently from a similar case in Germany (Henning et al., 2002). The *Waddliaceae* is one of eight families described to date, within the phylum *Chlamydiae* (Collingro et al., 2011; Taylor-Brown et al., 2015), all of which are obligate intracellular pathogens able to infect a variety of hosts covering much of the animal kingdom. The best known family of this phylum is the *Chlamydiaceae*, classical pathogens of humans and animals, some of which are known for their high zoonotic potential and ability to cross species borders, such as *Chlamydia psittaci*, the agent of psittacosis in birds and humans and *Chlamydia abortus*, an agent of fetal death and abortion in ruminants and humans (Longbottom and Coulter, 2003). *Waddlia chondrophila* may similarly pose a zoonotic risk, based on evidence from serological tests and quantitative real-time PCR in cases of human miscarriage and respiratory disease (Baud et al., 2007, 2011, 2014; Haider et al., 2008; Goy et al., 2009). A marked difference to the *Chlamydiaceae*, however, is the additional ability of *W. chondrophila* to infect and replicate in phagocytic cells, including macrophages and free-living amoebae, at least *in vitro* (Goy et al., 2008; Lamoth and Greub, 2010). This considerably complicates experiments to uncover the infection mechanisms and routes *W. chondrophila* uses *in vivo*, adding to the intrinsic difficulty of tracing infectious processes in whole animals. This knowledge is, however, key to the design and application of effective treatment strategies for the *Waddliaceae* in particular and for the *Chlamydiae* as a whole.

*In vitro* data indicates that *Waddlia* may be able to infect quite different hosts, widening the choice available when considering model organisms. The first cultivation of *W. chondrophila* was achieved in bovine turbinate cells and mouse macrophages (Dilbeck et al., 1990; Kocan et al., 1990). Subsequent *in vitro* studies showed that *W. chondrophila* is potentially a highly versatile pathogen, able to infect and replicate in McCoy cells, buffalo green monkey cells, human fibroblasts (Henning et al., 2002), Vero cells, human pneumocytes and endometrial cells (Kebbi-Beghdadi et al., 2011b), as well as in human macrophages (Goy et al., 2008). During the infection of macrophages, *W. chondrophila* avoids degradation by successfully preventing the fusion of the endosome with a lysosome and relocating in a vacuole expressing endoplasmic reticulum (ER) markers (Croxatto and Greub, 2010). Freshwater amoebae of the genus *Acanthamoeba* are also susceptible to infection with *W. chondrophila* (Lamoth and Greub, 2010). Furthermore, *W. chondrophila* was recently found to be able to invade and proliferate in two fish cell lines derived from fathead minnow (*Pimephales promelas*; EPC-175) and rainbow trout (*Oncorhynchus mykiss*; RTG-2) (Kebbi-Beghdadi et al., 2011a). In addition to mammalian hosts, *W. chondrophila* has been isolated from aquatic environments as diverse as sediments from the eastern Mediterranean Sea (Polymenakou et al., 2005) and freshwater samples from well water sources in Spain (Codony et al., 2012). According to these findings, it has been speculated that freshwater protists and fish could potentially serve as an aquatic reservoir for *W. chondrophila*, that one possible transmission route is water-borne and by inference, that fish may not only be a valuable alternative model but also a natural host.

Indeed, chlamydial disease affects both marine as well as freshwater fishes causing the disease epitheliocystis (Hoffman et al., 1969; Draghi et al., 2004; Meijer et al., 2006; Stride et al., 2014) in which bacteria-filled intracellular inclusions are found infecting gill and skin epithelia. Several chlamydial agents of epitheliocystis have been described so far, some of which are closely related to the *Waddliaceae* such as *Ca. Clavichlamydia salmonicola* (Karlsen et al., 2008), *Ca. Syngnamydia venezia* (Fehr et al., 2013) and *Ca. Syngnamydia salmonis* (Nylund et al., 2015), whereas others are more distantly related such as members of the deep rooted Piscichlamydia clade, *Ca. Piscichlamydia salmonis* (Draghi et al., 2004; Schmidt-Posthaus et al., 2012), *Ca.* Parilichlamydiaceae (Stride et al., 2013a), *Ca.* Similichlamydiaceae (Stride et al., 2013b,c; Steigen et al., 2015; Seth-Smith et al., this issue), and *Ca.* Actinochlamydiaceae (Steigen et al., 2013). In a ground breaking study, Lagkouvardos and colleagues discovered up to 181 new putative families of the *Chlamydiae* from primarily marine and fresh water sources, of which the *Waddliaceae* formed a prominent clade (Lagkouvardos et al., 2014).

Expanding genomic information has greatly improved our understanding of potential mechanisms underlying host diversity and disease pathology and is an essential step for establishing a model pathogen-host system. The availability of the *W. chondrophila* genome provided precious information on putative virulence factors of these bacteria (Bertelli et al., 2010), however, there is a great need to develop animal models to test the ideas coming from these efforts (Bachmann et al., 2014). Ideally, such an animal model would lend itself to high throughput screening and share an immune system with close similarities to that of humans and other animal hosts. As a model organism in infection biology, the zebrafish (*Danio rerio*) has become increasingly popular. Many infection systems using larval and adult zebrafish have been successfully developed over the past decade (Kanther and Rawls, 2010; Meijer and Spaink, 2011; Fehr et al., 2015). Its small size, ease of breeding, high fertility and genetic tractability combined with transparent larval stages combine to make it an attractive model organism for science. The zebrafish immune system displays many similarities to that of mammals, with counterparts for most of the human immune cell types (Meeker and Trede, 2008) and have conserved mechanisms for the recognition of microbes like Toll-like receptors (TLRs) (Hall et al., 2009). The zebrafish innate immune system starts to develop as early as 24 h post fertilization (hpf) with a population of primitive macrophages which derive from cells located in a region of the yolk sac near the heart (Herbomel et al., 1999). At 2 days post fertilization (dpf), subpopulations of macrophages can already be observed throughout the organism along with neutrophils whose development initiates between 32 and 48 hpf. The development of the adaptive immune system lags behind, with the first lymphocytes observed from 4 dpf, although fully developed adaptive immunity takes another 4 weeks to mature (Meijer and Spaink, 2011). For this reason, it is possible to exclusively observe the reaction of the innate immune system within an infection during the first week of larval development. Previous studies have proposed that recognition by TLRs could mediate an efficient immune reaction against chlamydial infection leading to bacterial clearance (Naiki et al., 2005), while in other cases TLR dependent recruitment of innate immune cells had an adverse effect by enhancing the bacterial load during an infection (Rodriguez et al., 2005). The activation of TLRs initiates an inflammatory response through signaling cascades that lead to cytokine production which promote recruitment of leukocytes to the infection site and phagocytosis of invading pathogens (Newton and Dixit, 2012). A key factor for TLR signal transduction is the downstream adaptor molecule MyD88, which interacts with all known TLRs and members of the interleukin-1 receptor (IL-1R) family, resulting in the induction of nuclear factor-kappaB (NF-kB) and mitogen-activated protein kinase (MAPK) signaling (Medzhitov et al., 1998; Warner and Nunez, 2013).

Zebrafish larvae have been used to study infections of many different bacterial pathogens like *Mycobacterium marinum, Salmonella typhimurium, Vibrio anguillarum, Listeria monocytogenes, Pseudomonas aeruginosa, Burkholderia cenocepacia, Staphylococcus aureus, Streptococcus iniae, Shigella flexneri*, and *Cronobacter turicensis* (Herbomel et al., 1999; Davis et al., 2002; van der Sar et al., 2003; O'Toole et al., 2004; Brannon et al., 2009; Clatworthy et al., 2009; Levraud et al., 2009; Vergunst et al., 2010; Adams et al., 2011; Prajsnar et al., 2012; Harvie et al., 2013; Mostowy et al., 2013; Fehr et al., 2015). However, none of these pathogens are obligate intracellular bacteria. By developing an infection model with *W. chondrophila*, we demonstrate the first zebrafish infection model for an obligate intracellular pathogen and member of the Chlamydiae. We established alternate routes of infection and analyzed preferred target cells for Waddlia infection *in vivo* and have taken initial steps to elucidate molecular mechanisms regulating infection, including the impact of MyD88 signaling in knockdown larvae.

## Materials and methods

### Zebrafish strains and husbandry

Zebrafish (*D. rerio*) strains used in this study were predominantly *albino* mutants (slc45a2^b4/+^) and transgenic fish of the *Tg(lyz:DsRED2)nz50* line that produce red fluorescent protein in cells of the myelomonocytic lineage able to migrate to inflammatory sites and phagocytose bacteria (Hall et al., 2007), primarily neutrophils from 50 h post fertilization (hpf) (Clatworthy et al., 2009). In addition, the *Tg(fli1a:eGFP)* line which produces green fluorescent protein in endothelial cells, was used to visualize the vascular system (Lawson and Weinstein, 2002). Adult fish were kept at a 14/10 h light/dark cycle at a pH of 7.5 and 27°C. Eggs were obtained from natural spawning between adult fish which were set up pairwise in separate breeding tanks. Embryos were raised in petri dishes with E3 medium (5 mM NaCl, 0.17 mM KCl, 0.33 mM CaCl_2_, 0.33 mM MgSO_4_) containing 0.3 μg/ml of methylene blue at 28°C. From 24 hpf, 0.003% 1-phenyl-2-thiourea (PTU) was added to prevent melanin synthesis. The *albino* mutants lack melanised melanophores, and for these PTU treatment was not necessary. Staging of embryos was performed according to Kimmel et al. (1995).

Research was conducted with ethics approval (no. 216/2012) from the animal research ethics committee of the Veterinary Office, Public Health Department, Canton of Zurich (Switzerland). Larvae were maintained up until 7 days post fertilization (dpf), at which time all were euthanized by applying an overdose of 4 g/l buffered tricaine (MS-222, Ethyl 3-aminobenzoate methanesulfonate, Sigma-Aldrich) in accordance with ethical procedures.

### Bacterial cultures

*Waddlia chondrophila* strain WSU 86-1044 (ATCC VR-1470) were grown within *Acanthamoeba castellanii* strain ATCC 30010 in 25 cm^2^ cell culture flasks containing 10 ml of peptone yeast extract glucose (PYG) broth (Greub and Raoult, 2002). Cultures were harvested after 5 days and filtered through a 5 μm membrane to remove remaining amoebae. The flow-through was centrifuged at 7000 g for 15 min. The resulting pellet of bacterial elementary bodies (EBs) was then suspended in E3 medium for bath immersion or phosphate buffered saline (PBS) for microinjection experiments. The infectivity of *W. chondrophila* was tested in Epithelioma Papulosum Cyprini (EPC) cells, to investigate the infection process and morphology of the bacteria in these fish epithelial cells (Supplementary Material Figure S1). Subsequently, Inclusion Forming Units (IFU) of the cultures were determined by infecting monolayers of EPC cells in 24-well plates with a 10-fold dilution series of 1 μl of the bacterial suspension at 28°C. After 24 h cells were fixed with 4% paraformaldehyde and subsequently stained with a primary rabbit anti-*Waddlia* antibody and detected with a secondary goat anti-rabbit-IgG conjugated to a fluorescent AlexaFluor dye. After imaging with a fluorescent microscope, inclusions were counted in Imaris (Bitplane) to calculate a mean IFU value.

### Bath immersion experiments

Zebrafish embryos between 24 hpf and 4 dpf were incubated in groups of 15 for each time point and each condition in 24-well plates with E3 medium containing 2 × 10^9^ IFU/ml of *W. chondrophila* at 28°C. Embryos younger than 48 hpf were manually dechorionated prior to immersion. After 4 h of incubation embryos were washed twice in fresh E3 medium and transferred to 6-well plates containing 4 ml of E3 medium per well and further incubated at 28°C. Embryos were then observed under a binocular microscope for signs of disease and survival twice a day. At several time points embryos from each group were euthanized in E3 medium containing 4 mg/ml buffered tricaine (MS-222).

### Microinjection experiments

For microinjections of *W. chondrophila* into zebrafish larvae bacteria were first harvested from a 5 days old amoebal co-culture as described above. The concentration of the *W. chondrophila* EBs was adjusted to 1000–2000 IFU/nl in PBS and 0.085% phenol red was added to visualize the injection procedure. Injections were done using pulled borosilicate glass microcapillary injection needles (Science Products, 1210332, 1 mm O.D. × 0.78 mm I.D.) and a PV830 Pneumatic PicoPump (World Precision Instruments). Prior to intravenious injections embryos of 2 dpf were manually dechorionated and anesthetised with 200 mg/l buffered tricaine (MS-222). Afterwards embryos were aligned on an agar plate and injected with 1 nl of the *W. chondrophila* suspension into the Duct of Cuvier, also known as common cardinal vein. Prior determination of the injected volume was performed by injection of a droplet into mineral oil and measurement of its approx. diameter over a scale bar. For swim bladder injections 4 dpf larvae were treated similarly but dechorionisation was not necessary because larvae were already hatched. 4 dpf larvae were then injected with a volume of 2 nl into the lumen of the swim bladder. After injections, infected larvae were allowed to recover in a petri dish with fresh E3 medium for 15 min. Subsequently, larvae were transferred in 6-well plates in groups of about 15 larvae in 4 ml E3 medium per well, incubated at 28°C and observed under a stereomicroscope twice a day. Samples for Immunofluorescence (IF), Transmission Electron Microscopy (TEM) and quantitative Polymerase Chain Reaction (qPCR) were taken at 0, 12, 24, 36, 48, 60 and 72 h post infection (hpi). Sampled larvae were euthanized with an overdose of 4 g/l buffered tricaine and transferred into different buffers and fixatives for subsequent analyses respectively.

### Whole mount immunofluorescence (IF) and histological stainings

For IF stainings, whole zebrafish larvae were fixed in 4 % paraformaldehyde at 4°C overnight followed by 100 % methanol overnight at −20°C. Samples were rehydrated in 50% methanol for 5 min and subsequently in H_2_O for 1 h before blocking in PBDT (PBS containing 1% BSA, 1% DMSO, 0.5% Triton X-100, 2.5% goat serum) for 6–8 h at room temperature. Larvae were then incubated with primary antibody overnight at 4°C. Primary antibody was detected by incubation with a secondary goat anti-IgG antibody conjugated to a fluorescent AlexaFluor dye (Life Technologies) at 4°C overnight. Additionally 4′-6-diamin-2-phenylindole (DAPI) was added to visualize bacterial and host DNA. Stained larvae were prepared for microscopy on objective slides mounted in 1.5% agarose, 50% glycerol and screened under a fluorescence microscope. Positive samples were subsequently screened in more detail for *W. chondrophila* inclusions by Confocal Laser Scanning Microscopy (CLSM).

For histological examination, whole zebrafish larvae were fixed in 4% paraformaldehyde at 4°C and embedded in cubes of cooked egg white in order to position them correctly for histological sections. These cubes containing the larvae were dehydrated in an ascending alcohol series ending in xylene and afterwards embedded in paraffin. Paraffin blocks were cut in 2–3 μm thin sections, mounted on glass slides and stained using a routine protocol for haematoxylin and eosin (HE) staining.

### Light-microscopy and image analysis

Overview images were taken with an upright light microscope (Olympus BX61) with both bright field and fluorescence modules. The fluorescence filter cube used was optimized for DAPI/FITC/TRIC. For higher resolution images, 3D-image stacks of whole mount samples were prepared using CLSM (Leica TCS SP5, Leica Microsystems). Various combinations of the fluorophors AlexaFluor dyes 594, 546, 488, GFP, dsRED, and DAPI were sequentially excited in descending series with the 594, 561, 488, and 405 nm laser lines, with emission signals collected within the respective range of wave lengths. Three-dimensional image stacks were collected sequentially (to prevent blue-green–red channel cross-talk) and according to Nyquist criteria and deconvolved using HuygensPro via the Huygens Remote Manager v2.1.2 (SVI, Netherlands). Images were further analyzed with Imaris 7.6.1 (Bitplane, Zurich, Switzerland) and aligned with Adobe Photoshop Elements 12. Fluorescent cells were quantified with the Imaris fluorescent spot counting tool.

### Transmission electron microscopy

For electron microscopy, larvae were fixed in a mixed solution of 1% paraformaldehyde and 2.5% glutaraldehyde in 0.1 M sodium phosphate buffer, pH 7.5 at 4°C overnight. Samples were prepared by embedding into epoxy resin and for TEM microscopy according to standard procedures. Epoxy resin blocks were screened for larval location using semithin sections (1 μm) which were stained with toluidine blue (Sigma-Aldrich) to visualize tissue. Ultrathin sections (80 nm) were mounted on copper grids (Merck Eurolab AG, Dietlikon, Switzerland), contrasted with uranyl acetate dihydrate (Sigma-Aldrich) and lead citrate (Merck Eurolab AG) and investigated using a Philips CM10 transmission electron microscope. Images were processed with Imaris (Bitplane) and assembled for publication using Adobe Photoshop.

### Quantitative PCR

A qPCR system was designed against the 16S rRNA gene from *W. chondrophila* (forward: 5′- AGTCCGGCTACACCAAGTATGC-3′, reverse: 5′-TGGCGAAGGCGGTTTTC-3′, probe: 5′-FAM-TTCGCTCCCCTAGCTTTCGGGCAT-TAMRA-3′), allowing quantification of the bacterial load of individual infected larvae. The TaqMan qPCR system was designed and validated against quantified serial dilutions of the target sequence cloned into the pCR2.1 plasmid and against total DNA extracts of non-infected larvae. Serial dilutions of pCR2.1 containing the target sequence were used as standards in each run. Total DNA of individual larvae was extracted with a MagNA Pure LC (Roche) robot and eluted in 100 μl elution buffer. Reactions were carried out on a StepOne Plus real-time PCR system (Applied Biosystems). TaqMan Fast Advanced reagents (Applied Biosystems) were used according to the manufacturer's instructions with 5 μl input DNA in a total reaction volume of 20 μl.

### Morpholino knockdown

Knockdown of MyD88 expression was done by standard microinjection of 1 nl of a 5 mM solution of anti-Myd88 morpholino (5′-GTTAAACACTGACCCTGTGGATCAT-3′, Gene Tools; Bates et al., 2007; Cambier et al., 2014) into the early zygote immediately after fertilization using borosilicate glass microcapillary injection needles (Harvard Apparatus, 30-0019, 1 mm O.D. × 0.58 mm I.D.) and an Eppendorf FemtoJet. A group of control larvae was injected with 1 nl of a control morpholino (5′-CCTCTTACCTCAGTTACAATTTATA-3′, Gene Tools) with no known target in zebrafish at the same concentration in parallel. Levels of MyD88 protein in morphant and control larvae were analyzed by Western Blot at 3, 4, and 5 dpf with a mouse anti-myd88 antibody, detected with a goat anti-mouse-IgG antibody conjugated to horseradish peroxidase (HRP).

## Results

### *W. chondrophila* is capable of infecting zebrafish larvae via bath-immersion

**Figure 1:**
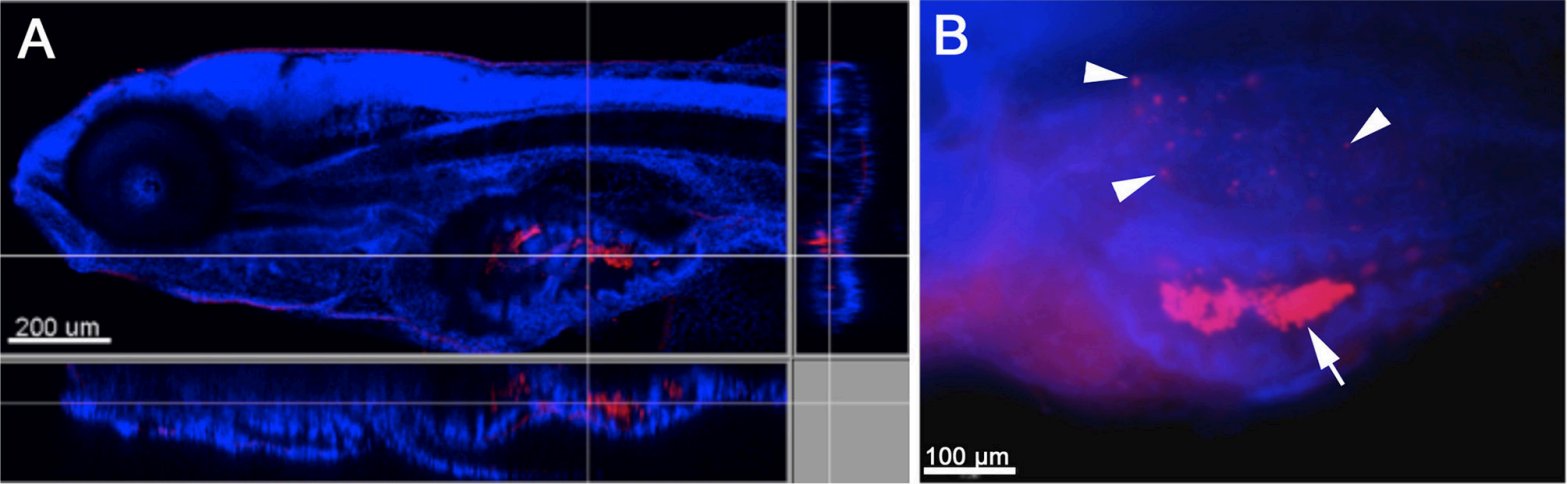
**Infection of zebrafish larvae with *W. chondrophila* via bath-immersion at 4 dpf followed by IF staining with an anti-*Waddlia* antibody (red) and DAPI (blue)**. Section view of a CLSM acquired 3D stack of a larva at 4 hpi. The section view exhibits a large amount of swallowed *W. chondrophila* inside the lumen of the intestine **(A)**. Fluorescence light-microscope appearance of the trunk region of a larva at 48 hpi shows in addition to the accumulation of *W. chondrophila* in the gut lumen (arrow), an infection of the swim bladder with bacterial inclusions (arrow heads) visible inside the epithelium **(B)**.

In order to first establish whether *W. chondrophila* can infect zebrafish through an oral or dermal route, embryos and larvae of different stages were incubated in a suspension of *W. chondrophila* infectious particles or EBs, in a bath-immersion experiment. While younger embryos and larvae of up to 72 hpf (hours post fertilization) stages could not be infected with this method, 4 dpf (days post fertilization) larvae appeared to swallow (Figure 1A) the bacteria and interestingly, succumbed to an infection of their swim bladder which we detected by IF staining (Figure 1B).

### Swim bladder infection can be provoked via microinjection

**Figure 2:**
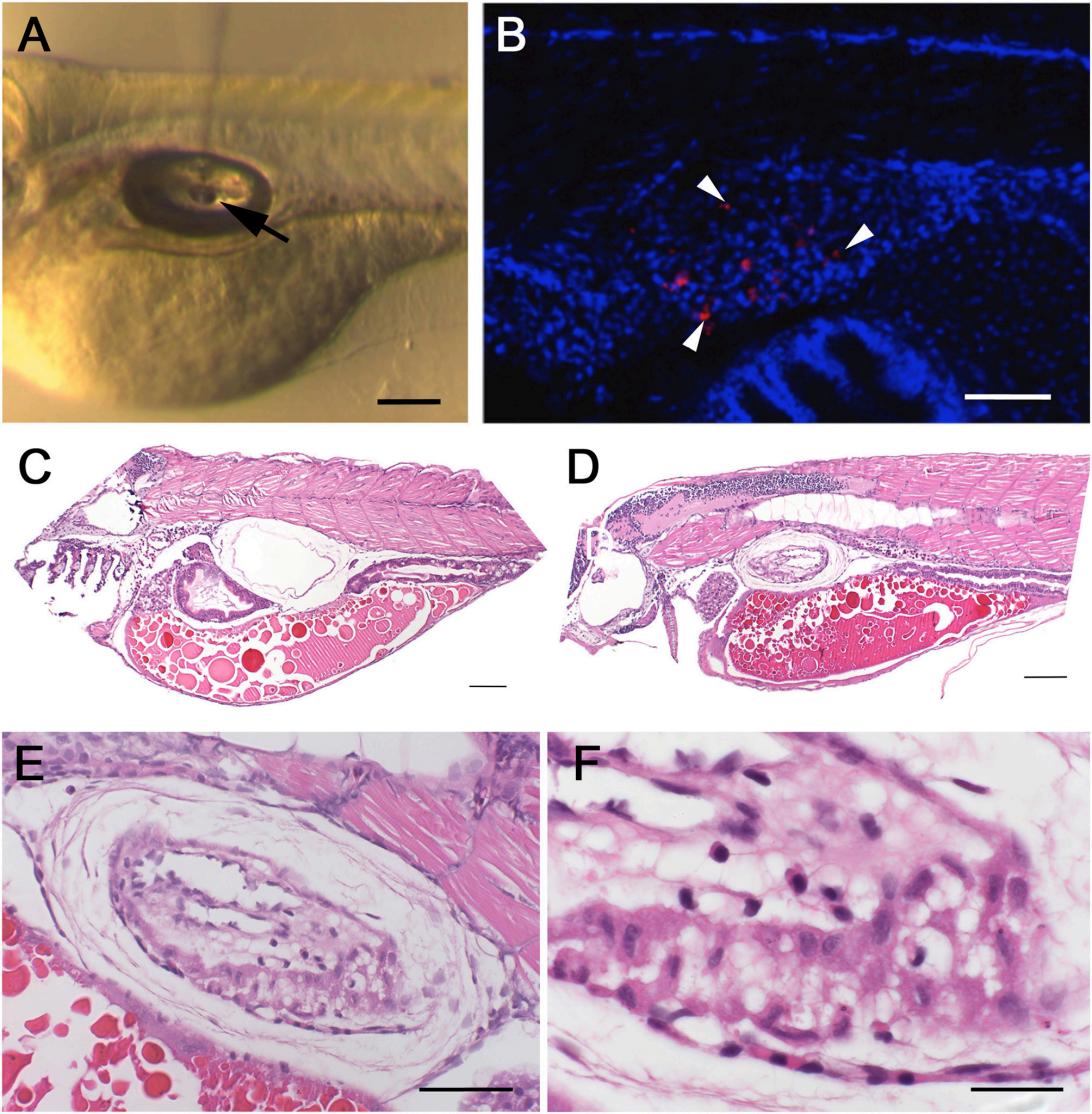
**Swim bladder infection in 4–5 dpf old larvae**. Microinjection of *W. chondrophila* EBs directly into the lumen of the swim bladder of 4 dpf larvae. A drop of approx. 1 nl of the bacterial suspension can be seen hanging on the tip of the injection needle (arrow) inside the air filled lumen of the swim bladder **(A)**. CLSM acquired 3D stack of the trunk region of a larva at 36 hpi, after IF staining with an anti-*Waddlia* antibody (red) and DAPI (blue). The swim bladder in the center of the image exhibits several bacterial inclusions (arrow heads) inside the epithelium **(B)**. Histology on HE-stained sections of the swim bladder of a PBS injected control larva **(C)** with normal appearance and of an infected larva **(D)** showing clear pathological changes, including thickening of the epithelium **(E)** and infiltration of innate immune cells like macrophages and neutrophils **(F)**. Scale bars **(A–D)** 100 μm, **(E)** 50 μm, and **(F)** 20 μm.

The swim bladder provides the only possibility to examine infections of air-exposed epithelium in fish and, as such, is a putative model for equivalent epithelia in humans, in particular the lungs. In this context, the spontaneous bath-immersion induced infection of the swim bladder and especially the attachment to the cilia of the epithelium was an exciting finding, although infection via bath immersion carried inherent experimental variability due to varying local concentrations of *W. chondrophila* and different gulping rates of individual larvae. Therefore, we tried direct microinjection of 10^3^ *W. chondrophila* EBs into the swim bladder lumen of 4 dpf larvae (Figure 2A). The resulting infection was comparable to that seen using bath-immersion but under more controlled conditions, confirming that directed injection can be used as a more reproducible method of establishing infection. In addition to live *W. chondrophila*, two groups of control embryos were injected with either heat-inactivated *W. chondrophila* or PBS in parallel. Samples were taken at 4, 24, 48, 72, and 96 hpi and screened for the presence of *W. chondrophila* inclusions by confocal laser scanning microscopy CLSM (Laser Scanning Microscopy) and TEM (Transmission Electron Microscopy). *W. chondrophila* inclusions were detected in the epithelium of the swim bladder as well as in adjacent tissues at 24–48 hpi (Figure 2B). In haematoxylin and eosin (HE) stained samples, the swim bladder walls of infected larvae were markedly thickened when compared with control animals (Figures 2C,D). The epithelial cells lining the cavity were piled up and were no longer a single cell row, with the cells themselves exhibiting a cuboidal instead of a flattened morphology (Figure 2E). The epithelium was surrounded by a moderate proliferation of fibroblasts resulting in a fibrosis, as has been described in mice for chlamydial lung infections (Jupelli et al., 2013). Neutrophils were also found to have migrated into the epithelial cell layers (Figure 2F). No increase in mortality was observed up until the end of the observation period of 3 days post infection (dpi) and infected larvae showed no altered behavior compared to control larvae.

### Intravenous microinjection of *W. chondrophila* causes a systemic infection

**Figure 3:**
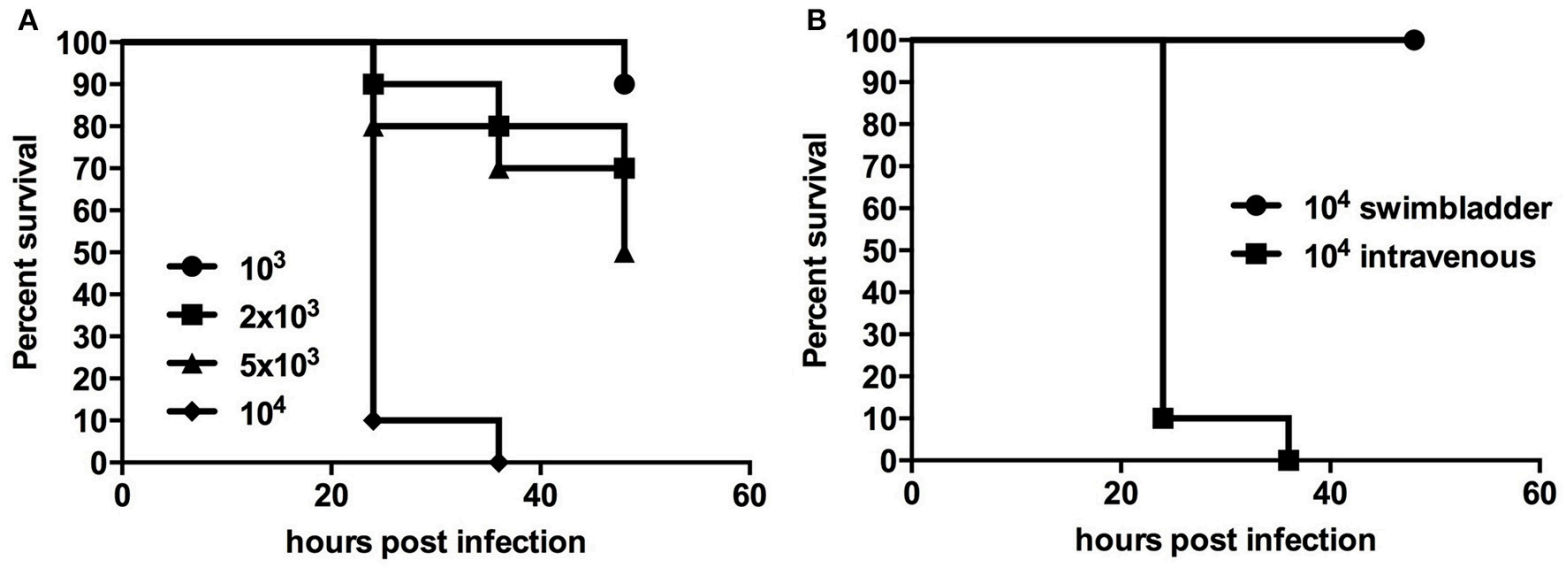
**Survival rates of larvae with a systemic infection after injection of different dosages of *W. chondrophila* show diminished survival with increasing dosage (A)**, while injection into the swim bladder has no impact on larval survival **(B)**.

**Figure 4:**
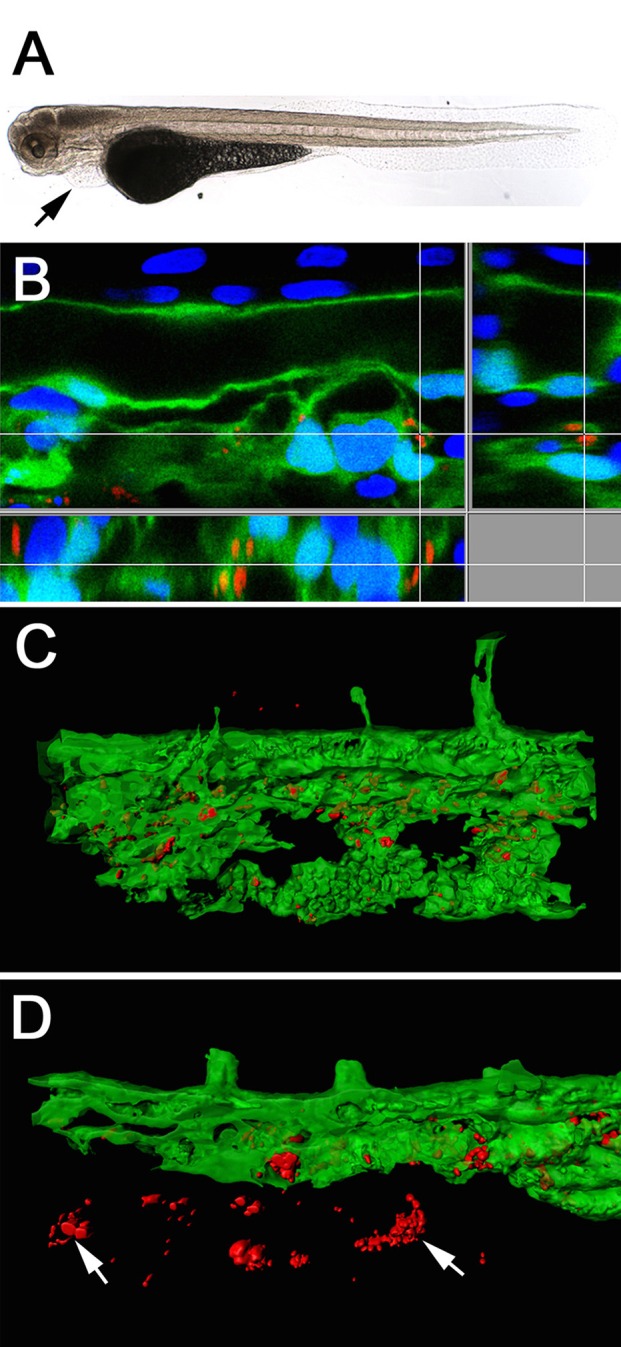
**Light-microscope appearance of a transgenic Tg(fli1a:eGFP) larva at 48 hpi after intravenous injection of live bacteria (A)**, showing an impaired blood flow ending in an almost completely stopped circulation, accompanied by pericardial oedema formation (arrow). Section view of a CLSM acquired 3D stack **(B)** after IF staining with an anti-*Waddlia* antibody, showing the infection of GFP expressing endothelial cells (green) with *W. chondrophila* (red). Host cell nuclei were stained with DAPI (blue). Surface rendering of the tail artery and caudal vein shows the distribution of the inclusions inside the vasculature at 24 hpi **(C)** and 36 hpi **(D)**, showing bacterial spread across endothelial bounds (arrows).

Chlamydial infection of vascular tissue has been an area of intense interest in the past, and not without controversy in respect to atherosclerosis (recently reviewed by Campbell and Rosenfeld (2014). It is also an aspect which is difficult to follow *in vivo* in other animal models but which is readily accessible in younger zebrafish larval stages by intravenous injection. We injected 48 hpf embryos intravenously via the Duct of Cuvier, also known as common cardinal vein with 10^3^–10^4^ *W. chondrophila* EBs. In comparison to the non-lethal swim bladder infection, a systemic infection caused a dosage dependent mortality rate of injected larvae (Figures 3A,B). The injection resulted in a rapid systemic infection with mortalities up to 100% within the first 24 h for the highest tested dosage (10^4^) and a LD_50_ within 48 hpi of approximately 5 × 10^3^. A dosage of 2 × 10^3^ *W. chondrophila* EBs was chosen for the following experiments to produce moderate mortalities of between 20 and 30% at 72 hpi. With that dosage larvae showed an increasingly impaired blood circulation between 24 and 48 hpi, resulting in the formation of a pericardial oedema (Figure 4A). Histologically, no tissue alterations could be seen.

To follow the distribution of *W. chondrophila* in the vascular system we used the transgenic Tg(fli1a:eGFP) line, which expresses green fluorescent protein in all endothelial cells. Tracking the infection with IF using an anti-*Waddlia* antibody, the maximal number of *W. chondrophila* inclusions were found at 36–48 hpi, distributed throughout the whole embryo, and with inclusions located both within the vasculature and beyond the bounds of the endothelium (Figures 4B–D). Cell types that were identified to be susceptible to *W. chondrophila* invasion during a systemic infection were predominantly endothelial cells and phagocytosing innate immune cells. Especially in regions where the blood is flowing more slowly, such as the fine capillary network of the tail muscles and veins, infection of the endothelium was more common.

### Morphology of *W. chondrophila* inside zebrafish host cells

**Figure 5:**
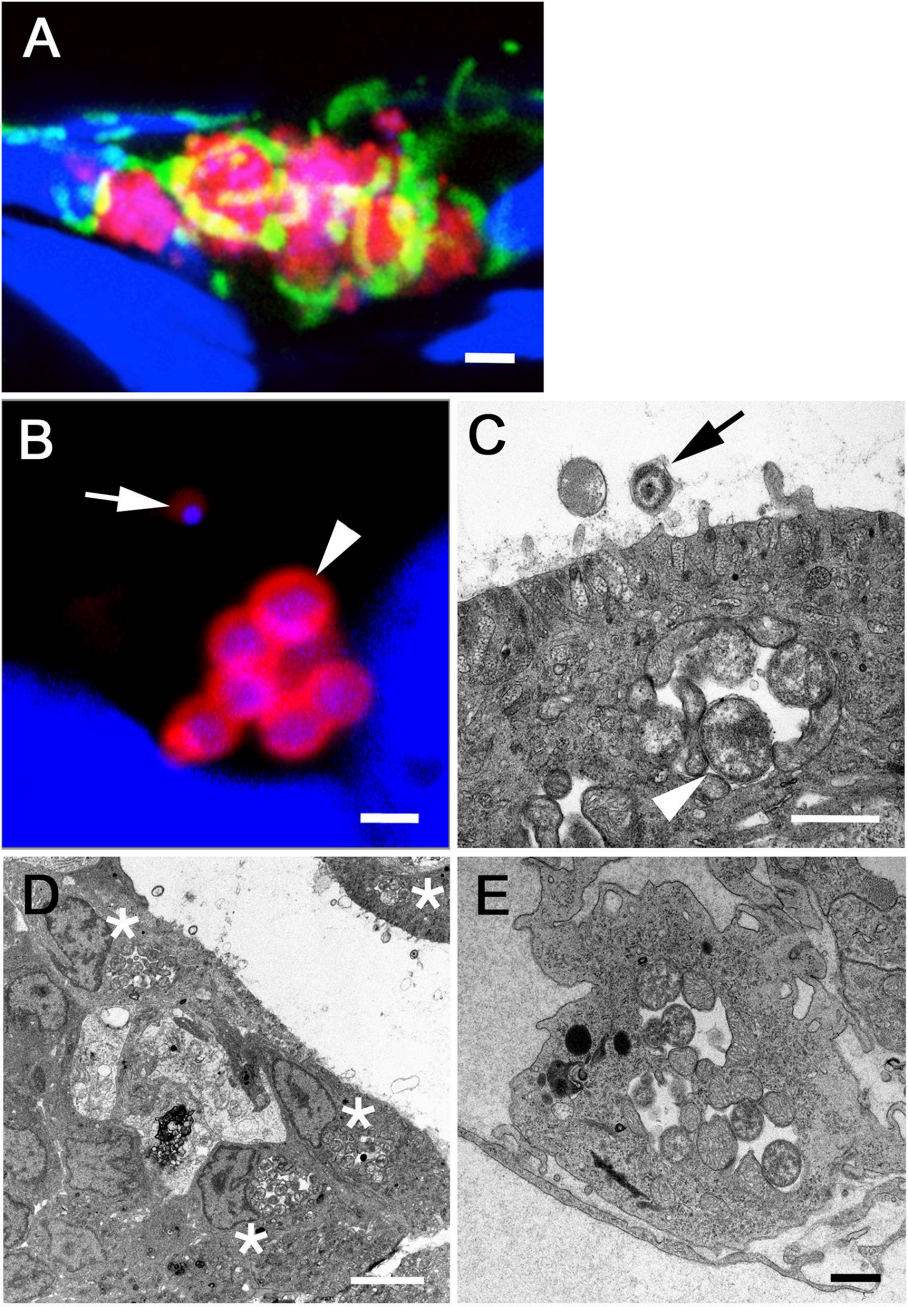
**CLSM acquired 3D image of *W. chondrophila* inclusions inside the swim bladder epithelium (A)** after IF staining of *Waddlia* (red), mitochondria (green) and DAPI staining of host cell and bacterial DNA (blue). The image shows the formation of several bacteria containing vacuoles inside epithelial cells, accompanied by the recruitment and close association with host cell mitochondria. Close-ups of single inclusions with CLSM **(B)** and TEM **(C)** reveal typical features of the chlamydial life cycle, such as transformation from the smaller infectious EB with condensed DNA (arrow) to the larger and metabolically active RB form with finely distributed chromatin (arrow head), dividing inside the vacuole. TEM images show also the morphology of infected epithelial cells of the swim bladder **(D)** and endothelial cells of blood vessels **(E)**, usually containing a single perinuclear inclusion (asterisks), strongly associated with host cell mitochondria. Scale bars **(A)** 2 μm, **(B,C,E)** 1 μm and **(D)** 5 μm.

To investigate the morphological features of *W. chondrophila* infection in zebrafish larvae, we performed detailed TEM and CLSM analyses of infected larvae after IF staining with an anti-*Waddlia* antibody and an anti-OxPhosIV antibody to stain mitochondria. *W. chondrophila* could be found to infect different cells of the zebrafish, predominantly epithelial cells of the swim bladder, phagocytes of the innate immune system and endothelial cells (Figure 5). The chlamydial inclusions inside these cells exhibited features typical for a *Waddlia* infection, including the transformation from the round shaped smaller (up to 0.5 μm in diameter) metabolically almost inactive but infectious EBs with highly condensed DNA, to the larger (about 0.9 μm in diameter) replicating reticulate bodies (RBs) with finely distributed chromatin inside a bacteria containing vacuole. Further, host cell mitochondria were readily recruited and closely associated with the inclusion, a characteristic for *Waddlia* infection *in vitro* (Croxatto and Greub, 2010) and now shown *in vivo*. While epithelial and endothelial cells usually contained a single perinuclear inclusion, individual phagocytes could harbor several inclusions of dividing bacteria. In TEM images of larvae that were injected into the swim bladder, some bacteria were in close relation to microvilli on the epithelial cell surface while others were found within the cytoplasm of these cells. The morphological structure of these bacteria on the surface resembled as well the typical morphology known for infectious particles of *W. chondrophila* (Rusconi et al., 2013). The perinuclear bacteria containing vacuoles inside epithelial cells were closely associated with host cell mitochondria and ER and are typical for actively replicating *W. chondrophila* observed *in vitro* (Rusconi et al., 2013).

### Innate immune reaction to *W. chondrophila* includes a strong recruitment of neutrophils

The zebrafish innate immune system reacts to *W. chondrophila* with a strong recruitment of inflammatory cells to the infection site, as we already observed in our histology analysis (Figure 2).

**Figure 6:**
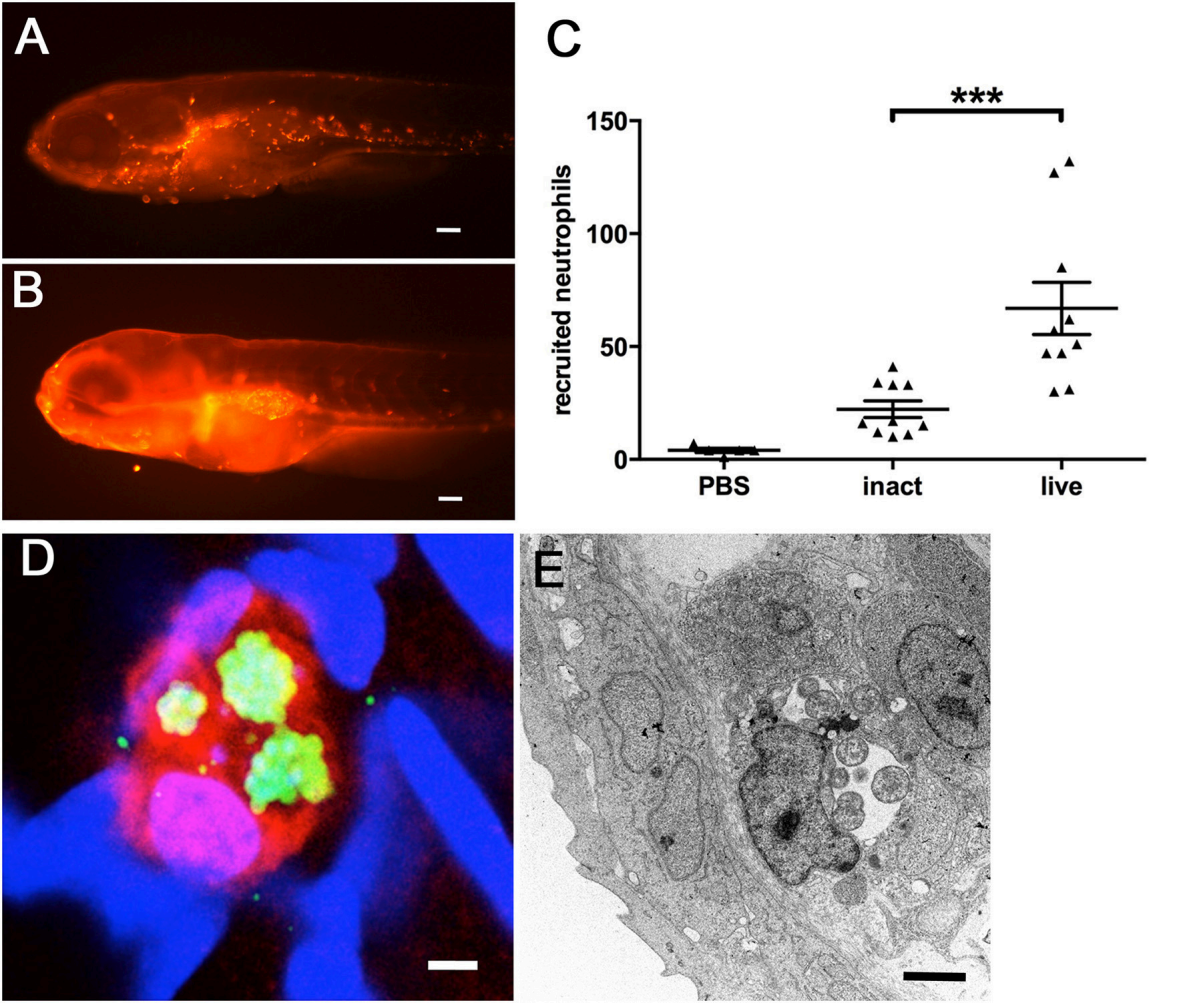
**Neutrophil recruitment and uptake of *W. chondrophila* monitored in transgenic Tg(*lyzC*:dsRed^nz50^) larvae at 8 hpi**. Whilst the swim bladder of a healthy larva is usually nearly devoid of innate immue cells **(A)**, the injection of *W. chondrophila* into the swim bladder activates strong neutrophil recruitment **(B)**. Quantification of recruited neutrophils **(C)** shows a significantly increased reaction to live *W. chondrophila* (live) compared to heat-inactivated (inact) bacteria or with sterile PBS injected control larvae, ^***^p < 0.001. After the uptake by a neutrophil *W. chondrophila* can successfully avoid its degradation and instead start replicating inside the phagosome to form an inclusion shown by 3D-CLSM **(D)** and TEM **(E)**. **(D)** shows a 3D acquired z-stack image of a dsRed expressing neutrophil (red) of the transgenic Tg(*lyzC*:dsRed^nz50^) line, harboring three *W. chondrophila* inclusions, visualized by antibody staining (green). DNA was stained with DAPI (blue). The TEM image in **(E)** shows a zebrafish phagocyte containing two inclusions of replicating *W. chondrophila* RBs. Scale bars **(A,B)** 100 μm, **(D)** 1 μm and **(E)** 2 μm.

This recruitment of inflammatory cells could be confirmed by using larvae of the transgenic Tg(*lyzC*:dsRed^nz50^) line, whose neutrophils express red fluorescent protein, easily assessable by fluorescence microscopy. Since the zebrafish swim bladder is an enclosed compartment, usually devoid of immune cells, we used our swim bladder infection assay in order to quantify the induced recruitment of neutrophils following microinjection. In these larvae, large numbers of neutrophils could be detected clustered inside the swim bladder (Figure 6B), compared to PBS injected control larvae (Figure 6A). We quantified the recruitment by counting the fluorescent neutrophils using the image analysis package of Imaris (Video S1). The results show further a significantly increased response of neutrophil invasion to live *W. chondrophila* compared to heat-inactivated bacteria (Figure 6C). Phagocytosed *W. chondrophila* show the ability to survive and replicate inside neutrophils (Figures 6D,E).

### Systemically infected larvae can be rescued by antibiotic treatment with tetracycline

*In vitro* studies have previously shown that *W. chondrophila* is susceptible to antibiotics of the tetracycline group, but is resistant to β-lactam antibiotics (Goy and Greub, 2009). Therefore we compared the effect of tetracycline and ampicillin on *W. chondrophila* infections *in vivo* by using our systemic infection assay, for which we compared the survival rates of differently treated larvae. In order to additionally quantify and compare the bacterial burden of individual larvae during the infection, we furthermore developed a specific quantitative PCR targeting the two-copy *W. chondrophila* 16S rRNA gene.

**Figure 7:**
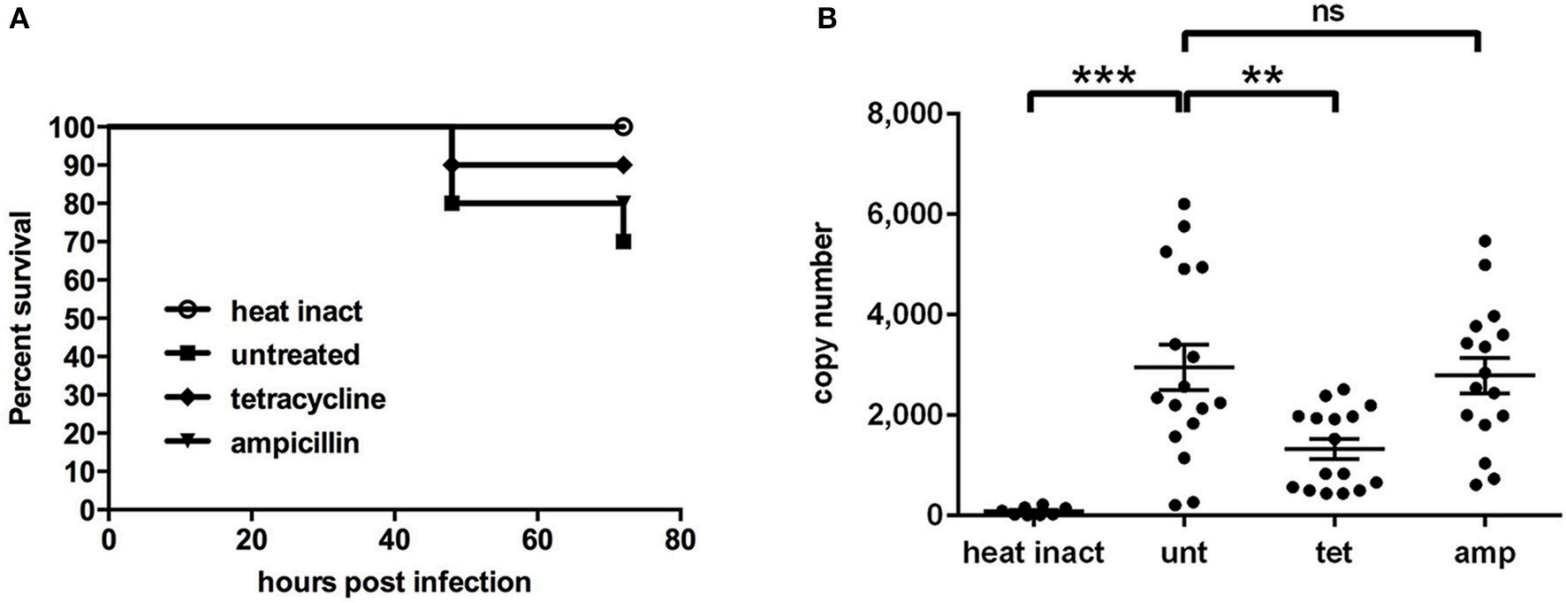
**Survival rates (A)** and bacterial load **(B)** are shown for larvae after intravenous injection of live *W. chondrophila* and subsequent treatment with 30 μg/ml tetracycline (tet) or 30 μg/ml ampicillin (amp) or left untreated (unt). Another group was injected with heat inactivated *W. chondrophila* (heat inact) for comparison. For survival rates **(A)** 10 larvae of each condition were observed in three independent experiments, respectively. The bacterial load **(B)** was determined at 36 hpi by qPCR of total DNA extracts from homogenates of individual larvae, targeting the *W. chondrophila* 16S rRNA sequence. Statistical analysis was done by one-way ANOVA with Bonferroni's posttest. ^***^p < 0.001, ^**^p < 0.01, ns = not significant. Mean values ± SEM are shown by horizontal bars.

While treatment with tetracycline significantly (*p* < 0.01) reduced *W. chondrophila* replication *in vivo* and increased the survival rate (Figure 7), treatment with ampicillin had a small but non-significant impact on the bacterial load compared to untreated larvae. Formation and distribution of *W. chondrophila* inclusions in untreated and ampicillin treated larvae were similar. In tetracycline treated larvae inclusion formation was strongly attenuated. Furthermore heat-inactivated bacteria were quickly cleared from the system.

### Effect of MyD88 on infection with *W. chondrophila*

**Figure 8:**
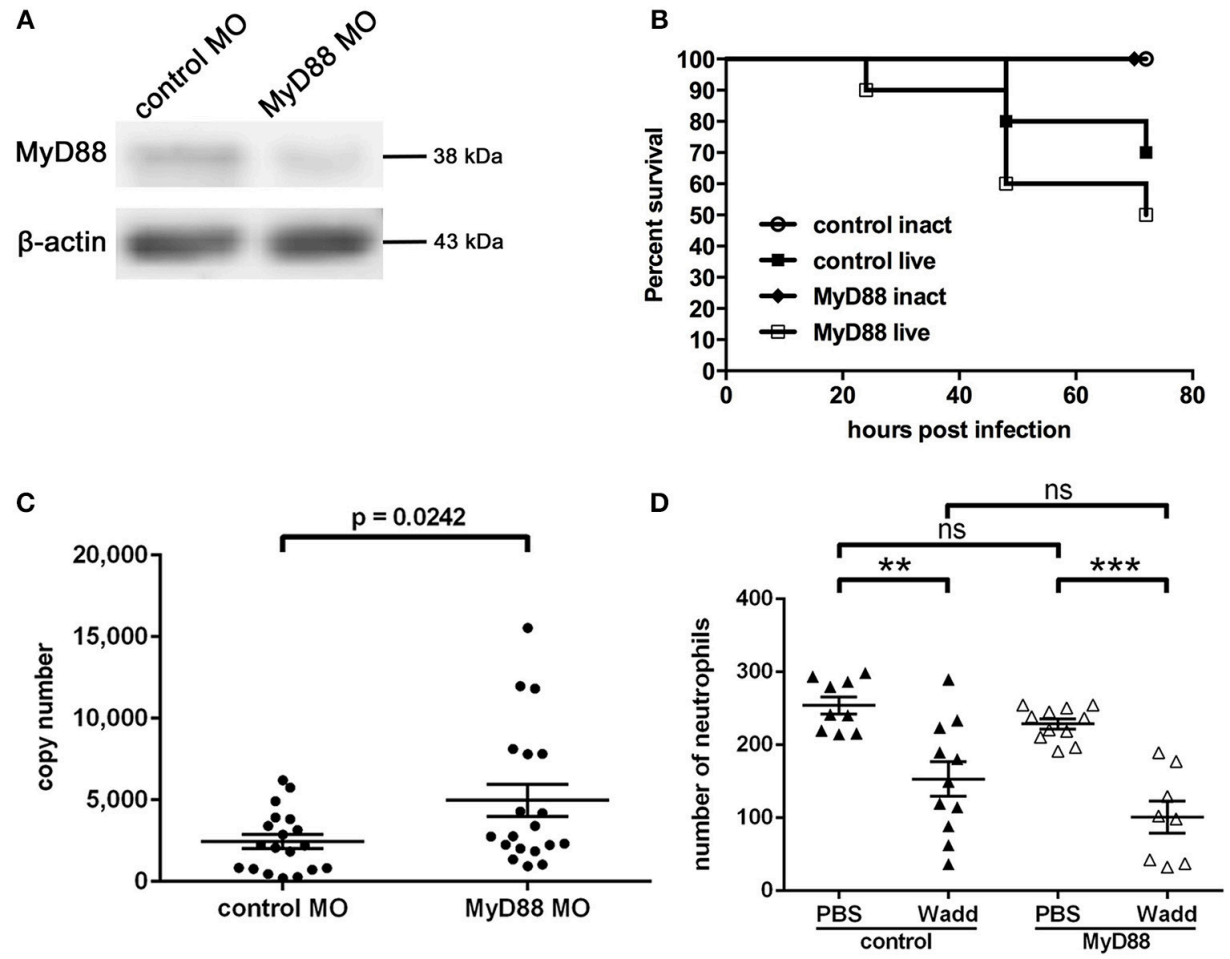
**Morpholino knockdown of MyD88, verified by Western Blot analysis (A)** of zebrafish MyD88 at 4 dpf, showing a depleted level of the protein in morphant fish. β-actin served as loading control. Survival rates of control and MyD88 depleted larvae at 36 hpi **(B)** show differences after intravenous injection of *W. chondrophila*. Morphant larvae show a slightly reduced survival rate compared to control larvae. Survival rates of 10 larvae for each condition were observed in three independent experiments, respectively. The bacterial load, determined by qPCR, **(C)** of MyD88 depleted larvae at 36 hpi is significantly (*p* = 0.0242) higher compared to control larvae. Statistical analysis was done by Student's unpaired *t*-test. Mean values ± SEM are shown by horizontal bars. Total numbers of neutrophils of whole individual control and MyD88 depleted larvae **(D)** were determined in the transgenic Tg(lyzC:dsRed)^nz50^ line at 36 hpi after intravenous injection of PBS or live *W. chondrophila* by counting dsRed expressing neutrophils after IF staining with an anti-dsRED antibody and subsequent CLSM analysis. The acquired 3D stacks of whole larvae were analyzed with the fluorescent spot counting tool in Imaris (Bitplane). The results show a significant depletion of neutrophils after injection of *W. chondrophila* compared to PBS injection in both normal and MyD88 depleted larvae with no significant differences between the two groups. Statistical analysis was done by one-way ANOVA with Bonferroni's posttest. ^***^p < 0.001, ^**^p < 0.01, ns = not significant. Mean values ± SEM are shown by horizontal bars.

The signaling molecule MyD88 has been shown to play a central role for pathogen recognition, immune reaction and survival of infected individuals during *Chlamydia* infection in several *in vivo* studies. Now we wished to address how MyD88 mediated signaling affects the infection with *W. chondrophila* in our zebrafish model. To investigate whether MyD88 signaling has a function during *W. chondrophila* infection in zebrafish, we knocked down MyD88 expression by injection of a specific morpholino at the 1-cell stage. The resulting knockdown lasted for up to 4 dpf as determined by Western Blot (Figure 8A). Survival rates and bacterial loads were compared between control and MyD88 knockdown larvae during systemic infection. Our results show, that MyD88 morphant larvae exhibited a 20% lower survival rate compared to control larvae with a similar initial injection dose of 2 × 10^3^ *W. chondrophila* EBs (Figure 8B). Furthermore, the bacterial load of MyD88 morphant larvae at 36 hpi was higher compared to control larvae (Figure 8C).

Additionally, to compare the total numbers of neutrophils during a systemic infection between morphant and control fish we injected transgenic Tg(*lyzC*:dsRed^nz50^) larvae either with sterile PBS, heat-inactivated *W. chondrophila* or live *W. chondrophila* (10^3^ EBs) at 2 dpf. Subsequently, total numbers of neutrophils within individual larvae were counted with the cell counting tool in Imaris. At 36 hpi, these numbers were found to be significantly depleted upon the systemic infection with *W. chondrophila* compared to PBS injected larvae, although no differences between MyD88 morphant and control fish were evident (Figure 8D).

## Discussion

We present the first zebrafish infection model for an obligate intracellular pathogen and member of the PVC superphylum, *W. chondrophila*, which produces a non-lethal swim bladder infection and a lethal systemic infection in zebrafish larvae. Primary target cells are epithelial cells of the swim bladder, endothelial cells of the vascular system and innate immune phagocytes. Moreover, in this study, we show for the first time that *W. chondrophila* can successfully survive and grow within zebrafish neutrophils. By using zebrafish larvae between 2 and 5 dpf our model provides the opportunity to study specifically the reaction of the innate immune system to a *W. chondrophila* infection.

The zebrafish swim bladder is an air-exposed epithelium, regarded as a homologous organ to the mammalian lung, having similar developmental and molecular ontogeny (Winata et al., 2009; Flores et al., 2010). Oral uptake of *W. chondrophila* by 4 dpf larvae results in a swim bladder infection, which can be reproducibly replicated by direct microinjection of *W. chondrophila* into the swim bladder. Since *W. chondrophila* has been linked to cases of pneumonia in humans (Haider et al., 2008), as have related organisms such as *Chlamydia pneumoniae* and *C. psittaci*, our model could provide a new approach to investigate aspects of human respiratory disease caused by these pathogens *in vivo*. Even more we could demonstrate that *W. chondrophila* enters the swim bladder epithelial cells and goes into the life cycle typical for these organisms forming metabolic active RB as described in mice with *C. pneumoniae* infections. A fibrosis surrounding the swim bladder was found to develop within few days after inoculation (Jupelli et al., 2013). Similarly to experimental infections of rodent lungs with other members of the order chlamydiales (Roger et al., 2010), the injection of live *W. chondrophila* into the swim bladder causes a strong recruitment of neutrophils to the infection site, compared to lack of neutrophil recruitment when using heat-inactivated bacteria. While zebrafish macrophages are known to react to invading bacteria at all locations, neutrophils are able to phagocytise surface associated bacteria in a vacuum-cleaner-like manner (Colucci-Guyon et al., 2011). Thus from our findings, it is possible that live *W. chondrophila* EBs adhere efficiently to the epithelium of the swim bladder, while heat-inactivated bacteria are unable to do so. The adhesion of live bacteria could lead to an increased neutrophil recruitment to the infection site.

Intravenous injection of *W. chondrophila* into zebrafish larvae leads to a strongly impaired blood circulation and subsequent oedema formation, as is typical for severe systemic inflammatory states such as sepsis, with the ensuing increased mortality correlating with increased dosage. This reaction was only provoked by injection of live infectious EBs and not by the use of heat-inactivated *W. chondrophila*. The infection triggers a strong innate immune response, evidenced by the initial recruitment and subsequent depletion of phagocytes. In addition to macrophages, we show for the first time that epithelial cells and neutrophils are among the preferred target cells for infection by *W. chondrophila in vivo*. The involvement of neutrophils in the host response to *W. chondrophila* was unexpected and is a new finding of this study which will stimulate a re-evaluation of the relative importance of macrophages and neutrophils in the initial clearance of chlamydial infections. Infection of endothelial cells has already been shown *in vitro* for *Chlamydia pneumoniae* (Godzik et al., 1995; Gaydos et al., 1996), suggesting at the time that these chlamydial pathogens may play a possible role in the development of vascular disease. Our findings with intravenously injected *W. chondrophila* now provide *in vivo* evidence to support this idea with another chlamydial pathogen and by using the zebrafish, a readily tractable model for future investigations.

*W. chondrophila* was first proposed as an abortifacient agent in ruminants (Dilbeck et al., 1990; Henning et al., 2002; Dilbeck-Robertson et al., 2003) and more recently in humans (Baud et al., 2007, 2011, 2014; Haider et al., 2008; Goy et al., 2009; Goy and Greub, 2009). The pathological mechanisms for this are unknown, although other chlamydial abortifacient agents are thought to act via a permanent infection of the intestine and from there via sepsis infecting the placenta and fetus. Aborted placentas of ewes after infections with *C. abortus* show, in addition to large areas of necrotic trophoblasts, a severe necrotizing vasculitis (Buxton et al., 2002). That *W. chondrophila* is able to establish an infection within vessel endothelium is a new finding and offers a potential mechanism to promote invasion of the placental tissues from the blood circulation.

Treatment of the infected larvae with tetracycline significantly reduces the bacterial load and increases the larval survival rate, while treatment with ampicillin is ineffective. Nevertheless, even without antibiotic treatment, the bacterial load starts to decrease after 72 hpi in surviving larvae. The infection is greatly reduced by 4 dpi although isolated inclusions or infection loci can remain. This experimental zebrafish model may thus be used to test new antibiotics *in vivo* during the initial infection window and may be used to investigate the effect of anti-virulence compounds such as Type III secretion system (T3SS) inhibitors, recently shown to be effective *in vitro* (Bertelli et al., 2010).

Our results show that although phagocytes are susceptible to infection with *W. chondrophila*, the innate immune system on its own is able to mount an effective initial counter strike against *W. chondrophila* infection in surviving larvae. Furthermore, we found that MyD88 mediated signaling contributes to an increased survival and a reduced bacterial load in control larvae compared to MyD88 morphants. These findings indicate a possible role of MyD88 dependent activation and recruitment of innate immune cells, including neutrophils, for an efficient reaction to *W. chondrophila* infection. On the other hand, we could not observe differences in phagocyte depletion between control and morphant larvae, which indicates that recruitment and phagocytosis of *W. chondrophila* could basically also be mediated by a MyD88 independent manner. MyD88 mediated signaling has also been found to contribute to the generation of an effective early immune response and increased host survival in a mouse model for *Chlamydia pneumoniae* infection (Naiki et al., 2005). Furthermore, it has been shown that *Parachlamydia acanthamoebae* is recognized and internalized by macrophages in a MyD88 independent manner (Roger et al., 2010), while on the other hand in another mouse model it was shown that neutrophil recruitment to *Chlamydia pneumoniae* infection is strongly depending on MyD88 signaling, although in this case, recruitment of neutrophils initially increased the bacterial load (Rodriguez et al., 2005).

The potential role of MyD88 signaling at later time points of an infection needs to be further investigated. Possible subsequent recognition of the *W. chondrophila* bacteria containing vacuole inside infected phagocytes by endosomal TLRs or cytoplasmic nucleotide binding oligomerization domain (NOD)-like receptors acting in association with MyD88 mediated signaling could lead to a more efficient response of the innate immune system to *W. chondrophila* infection. An essential role for the recognition and defensive reaction induced by NOD-like receptors has already been shown for *Chlamydia pneumoniae* and *Chlamydia trachomatis* (Buchholz and Stephens, 2008; Shimada et al., 2009). Whether *W. chondrophila* is also recognized by intracellular pattern recognition receptors is another key aspect for future studies.

Taken together, our results complement those from mouse models, but offers new insights into pathogenesis and immune response during *Chlamydia* infections. In particular, the use of different injection sites permits a staged analysis of separate events in an infection and the opportunity to understand the molecular mechanisms guiding these processes. The genetic tractability of the model is a particularly opportune addition to the tools available to the *Chlamydia* field, being potentially high throughput for screening of novel anti-chlamydial agents and especially useful given the recent progress in development of transgenic *Chlamydia* (Gérard et al., 2013; Song et al., 2013). The zebrafish model presented here offers chlamydial researchers in particular, and the PVC field in general, a powerful new experimental tool.

## Author contributions

AF, AL, AV, HS, LV, MR, PC, and SN conceived and designed the experiments. AF, AL, HS, LN, LV, MR, and SN performed the experiments. AF, AL, AV, GG, HS, LN, LV, MR, PC, and SN analyzed the data. AF, GG, HS, LV, MR, and PC wrote the manuscript.

## Funding

This study was supported by the EU through Marie Curie IEF grant number 332058, the SBF through COST Action 867: Fish Welfare in European Aquaculture and by the Swiss National Science Foundation (SNF), grant number 310030_138533/1. The funders had no role in study design, data collection and interpretation, or the decision to submit the work for publication.

### Conflict of interest statement

The authors declare that the research was conducted in the absence of any commercial or financial relationships that could be construed as a potential conflict of interest.
