# Supplementary material for: A Zebrafish Model for Chlamydia Infection with the Obligate Intracellular Pathogen Waddlia chondrophila
Source: Front Microbiol. 2016 Nov 18;7:1829. doi: 10.3389/fmicb.2016.01829 (PMC5114312; doi:10.3389/fmicb.2016.01829)
Supplement: Supplementary file 2 [file Presentation1.PDF]

Supplementary Material

A zebrafish model for *Chlamydia* infection with the obligate intracellular pathogen *Waddlia chondrophila*

Alexander G. J. Fehr[1,#], Maja Ruetten[1+], Helena M. B. Seth-Smith[1,2,§], Lisbeth Nufer[1], Andrea Voegtlin[3,†], Angelika Lehner[4], Gilbert Greub[5], Philip S. Crosier[6], Stephan C. F. Neuhauss[7] and Lloyd Vaughan[1,*]

[1]Institute for Veterinary Pathology, Vetsuisse Faculty, University of Zurich, Zurich, Switzerland
[2]Functional Genomics Center Zurich, Molecular and Life Sciences, University of Zurich, Zurich, Switzerland
[3]Institute of Veterinary Bacteriology, Vetsuisse Faculty, University of Zurich, Zurich, Switzerland
[4]Institute for Food Safety and Hygiene, Vetsuisse Faculty, University of Zurich, Zurich, Switzerland
[5]Institute of Microbiology, University Hospital Center and University of Lausanne, Lausanne, Switzerland
[6]Department of Molecular Medicine and Pathology, School of Medical Sciences, University of Auckland, Auckland, New Zealand
[7]Institute of Molecular Life Sciences, University of Zurich, Zurich, Switzerland

* Corresponding author: Lloyd Vaughan, Institute for Veterinary Pathology, Vetsuisse Faculty, University of Zürich, Switzerland. Email. vaughanl@vetpath.uzh.ch

Supplementary Data

Video S1. Neutrophil recruitment to the swimbladder. CLSM acquired 3D stack of the swimbladder of a Tg(lyzC:dsRed)^{nz50} larva at 8 hpi. Neutrophil recruitment was quantified with the cell counting tool in Imaris (Bitplane). Each spot (red) represents a single cell (yellow). DNA was counterstained with DAPI (blue) to define the region of interest (swimbladder).
