# Supplementary material for: A Zebrafish Model for Chlamydia Infection with the Obligate Intracellular Pathogen Waddlia chondrophila
Source: Front Microbiol. 2016 Nov 18;7:1829. doi: 10.3389/fmicb.2016.01829 (PMC5114312; doi:10.3389/fmicb.2016.01829)
Supplement: Supplementary file 3 [file DataSheet1.docx]

**Figure S1. Infection of EPC cells with *W. chondrophila***

**(A-C)** TEM of *W. chondrophila* EBs, freshly harvested from an amoeba co-culture and injected into eggwhite. **(D)** TEM of non-infected EPC cells. **(E)** TEM of EPC cells, infected with *W. chondrophila.* **(F)** TEM of a *W. chondrophila* inclusion, containing replicating RBs and surrounded by host cell mitochondria. **(G-I)** IF staining of infected EPC cells with concanavalin A (red), an anti-Waddlia antibody (yellow), an anti-OxPhosIV antibody to stain mitochondria (green) and DAPI. The perinuclear BCVs show close association with host cell mitochondria. **(I)** DAPI and anti-OxPhosIV antibody alone.
