# Supplementary figures and images for: A Zebrafish Model for Chlamydia Infection with the Obligate Intracellular Pathogen Waddlia chondrophila
Source: Front Microbiol. 2016 Nov 18;7:1829. doi: 10.3389/fmicb.2016.01829 (PMC5114312; doi:10.3389/fmicb.2016.01829)

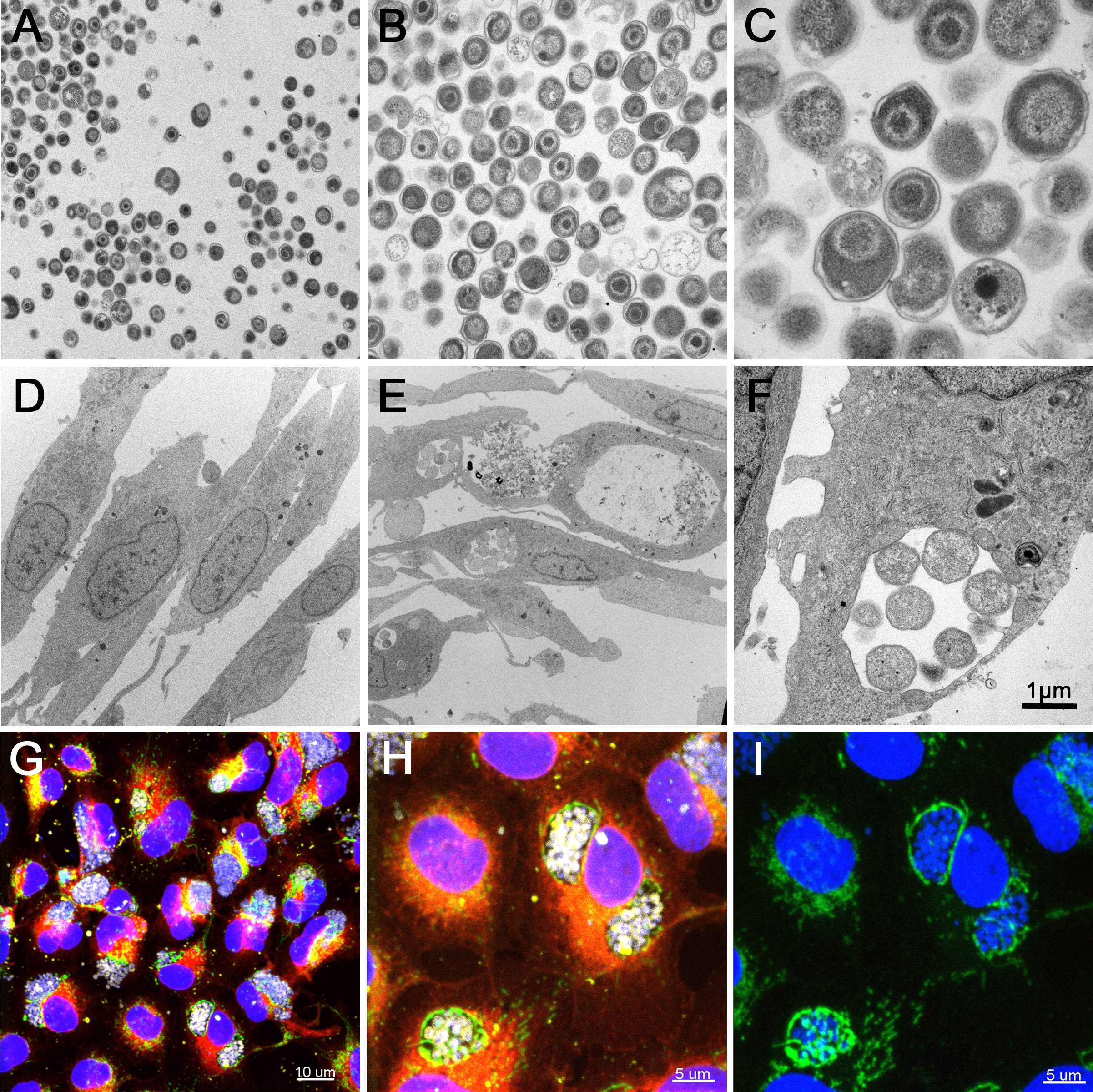

Supplement: Supplementary file 4 [file Image1.jpg]
